# Developing and validating the self-transcendent emotion dictionary for text analysis

**DOI:** 10.1371/journal.pone.0239050

**Published:** 2020-09-11

**Authors:** Qihao Ji, Arthur A. Raney

**Affiliations:** 1 School of Communication & the Arts, Marist College, Poughkeepsie, NY, United States of America; 2 School of Communication, Florida State University, Tallahassee, FL, United States of America; University of Zurich, SWITZERLAND

## Abstract

Recent years have seen a growing amount of research effort directed toward what positive media psychologists refer to as self-transcendent emotions, such as awe, admiration, elevation, gratitude, inspiration, and hope. While these emotions are invaluable to promote greater human connectedness, prosociality, and human flourishing, researchers are constrained in terms of analyzing self-transcendent emotions as expressed in spoken and written languages. Drawing upon the word-counting approach of the text analysis paradigm, this project aimed at constructing a dictionary tool—Self-Transcendent Emotion Dictionary (STED)—which can be uploaded into mainstream, text analytic software (e.g., LIWC) to identify and analyze self-transcendent emotions in large corpora. This dictionary tool was then refined and validated via three studies, where individual words were first rated with regard to their fitness into the proposed construct (Step 1), and then used to analyze essays written to reflect the corresponding construct (Step 2). Finally, the refined dictionary was applied to examine words used in nearly 4,000 human-coded *New York Times* articles (Step 3). Results indicated that the final dictionary, consisting of 351 lexicons and phrases, exhibits acceptable face and construct validity, and possesses a reasonable level of external validity and applicability. Despite its shortcoming in accounting for the rhetorical techniques ingrained in natural human language, the STED could be instrumental for social scientific inquiry of positive emotions in textual narratives.

## Introduction

In recent years, a growing amount of research effort has been directed toward what psychology, communication, and media scholars refer to as self-transcendent emotions [1–6]. These emotions, while diverse in their manifestations, share certain common characteristics in their effects on individuals. More precisely, these emotions can lead to increased human connectedness, prosociality, human flourishing, and general subjective well-being [6–8]. A wide range of media content has been associated with the elicitation of self-transcendent emotions, ranging from news articles (e.g., Upworthy, Huffington Post’s Good News) to TV programs (e.g., *30 for 30*, *This is Us*), movies (e.g., *Hidden Figures*, *The Pursuit of Happyness*), and songs (e.g., *Imagine* by John Lennon, *Everybody Hurts* by REM) [2, 9]. Further, audiences report routinely experiencing self-transcendent emotions with such content; according to one recent national survey, more than 90% of American adults reported having been recently inspired by media [9].

A series of content analyses has identified specific depictions that can elicit self-transcendent emotions in audience members, such as the appearance of nature, expressions of religious beliefs, and an appreciation of moral virtue [2]. The resulting manual content-coding scheme has enabled researchers to identify the presence/absence of self-transcendent emotions (and the corresponding elicitors) across a variety of visual and audiovisual media [2, 6]. Despite the value and utility of such a coding scheme, media researchers face the challenge of understanding the *degree* to which specific portrayals convey various self-transcendent emotions. Meanwhile, in the era of big data, many communication researchers have also expressed the necessity to develop computer-assisted content analytic strategies for processing large volumes of data both efficiently and accurately [10, 11]. Coupled with the fact that much of the modern media content is still created (e.g., personal diaries, books, many social media posts) or can be transformed into textual formats (e.g., TED talks, transcripts of podcasts, etc.), the effort for developing a tool that enables researchers to quickly identify the implied self-transcendent emotions in textual narratives seems warranted.

Extensive research in psychology has shown that word use and linguistic features can reflect an individuals’ thoughts, emotions, and feelings, and thus can be used to identify people’s social and psychological states [12–14]. Building upon that premise, in this research, we report on the development and validation of a computerized, content-analytic tool—the Self-Transcendent Emotion Dictionary (STED)—that can help researchers identify terms associated with self-transcendent emotions in text-based media content (e.g., news articles, speech, books, song lyrics), so as to understand the expressions of those emotions embedded in spoken and written languages.

Designing such a tool naturally comes with many trade-offs. For one, the STED is devised under the premise of the word-counting system; as such, the lists of words and phrases only resemble our best approximation for each self-transcendent emotion (outlined below) at the moment. And just as any other tool for text analysis, continuous revision of the STED is indispensable as knowledge of transcendence-related processes evolves.

## Self-transcendent emotions

According to the positive and moral psychology literature, transcendence is considered one of the most important human virtues and character traits valued across all cultures [15]. Specifically, transcendence is the dispositional trait to strive for and connect with purpose and meaning greater or higher than ourselves and is made manifest through various behaviors (or character strengths) including an appreciation of beauty and excellence, gratitude, hope, humor, and spirituality. Those behaviors are typically accompanied by *self-transcendent emotional experiences* [5]. For instance, appreciating the beauty of the universe on a starry night can generate a sense of awe; holding a strong faith in a better future and working towards that can promote feelings of hope. Further, due to basic empathic processing, seeing—or in our case, reading about—someone displaying transcendence-related behaviors or experiencing self-transcendent emotions can trigger self-transcendent emotional experiences in the witness. Thus, by encountering others in transcendence-related situations, we too can experience self-transcendent emotions (and, in turn, further develop our own trait transcendence).

Self-transcendent emotions are thought to be a unique collection of positive emotions in that they do not primarily concern the person experiencing them but rather direct their focus and attention to others. They are typically elicited by witnessing goodness or virtue found outside of ourselves, often in other people and their actions [3, 4, 16, 17]; in turn, they also are thought to motivate altruism, connectedness, and well-being. Throughout the literature, positive psychologists have identified and discussed five prototypical self-transcendent emotions: awe, elevation, admiration, gratitude, and hope [2, 3, 5, 9]. Additionally, in light of the growing emphasis on inspiration as a distinctive positive emotion [17], this study also approached inspiration as one specific self-transcendent emotion which overlaps with the five prototypical ones detailed hereafter.

Awe is a complex, compound emotion, typically described using words such as *wonder*, *amazement*, and *fascination*; it is characterized by the appraisal of something massive (vastness) that requires cognitive adjustments (accommodation) in light of new experience [18]. Awe can be elicited by a range of social and natural stimuli, such as witnessing a display of great skills or standing in front of the Grand Canyon [18, 19]. Elevation is often described using terms such as *uplifting*, *moving*, and *warm*; it is often evoked in conjunction with love [8, 16] and can be experienced when one encounters behavioral manifestations of moral beauty and humanity’s better nature, such as helping others in need, acting out loyalty and gratitude, and caring for children [16, 18]. Admiration is a similar emotional experience but is typically activated by encountering *non-moral* excellence, extraordinary skill, talent, or achievement.

Gratitude and hope have received relatively less attention by moral psychologists than awe, elevation, and admiration. Conceptually, gratitude is characterized by the sense of wonder, thankfulness, and appreciation for life in response to either someone doing something for you or simply toward the positive things in life [20]. It can be elicited when we perceive that someone else or another source (e.g., the divine, luck, fate) has intentionally improved our well-being. Finally, hope is the feeling associated with a belief that things can change for the better, whether in our own lives, in the lives of others, or for the world [4]. Hope motivates us to change things, inspires us to do better, and energizes us to act [21].

Another psychological construct that is closely relevant to the self-transcendent emotions is inspiration. Thrash and Elliot [22] argued that inspiration entails three dimensions: transcendence, evocation, and motivation. First, inspiration involves awareness of something that transcends our ordinary concerns and that is intrinsically and highly valued. Second, inspiration is evoked or awakened by an influence (e.g., person, situation, action) outside of one’s self; that is, we do not inspire ourselves. Finally, inspiration involves the motivation to express or actualize the new insight or idea; in psychological terms, this motivation is approach-oriented. Thrash and Elliot [23] demonstrated that inspiration as an emotional state can be meaningfully discriminated from positive affect. A recent study further categorized inspiration as a specific type of self-transcendent emotions [17]. Considering these works, we conceptualized inspiration as a general transcendence-related emotion, sharing the fundamental quality of the five self-transcendent emotions (i.e., transcendence) discussed above but also representing broader concerns than each of the five emotions in its scope. As such, it is subsequently referred to as “general inspiration” to signify it inclusive nature.

## Computerized text analysis and LIWC

As noted earlier, many psychology and communication researchers have long argued that word usage reflects people’s emotional and cognitive processes. According to Tausczik and Pennebaker [24], modern text analysis can be traced back to the early days of psychology, where psychiatrists would use it as a way to detect what was regarded as people’s hidden thoughts and intentions, as well as to diagnose mental-health issues. Early strategies involved, for instance, having physicians analyze each clause of a sentence uttered by individuals [25] or having trained raters read and rate a corpus of texts or transcripts based on words and phrases that represent various psychological dimensions [24, 26]. More recently, however, growing interests and efforts have been devoted to the seemingly more objective word-counting approach, which assumes that general social and psychological properties can be reflected with specific words as used in given corpora. Notably, the acceptance of word-counting strategy is not granted without careful scrutiny [27]. In particular, many have argued that the word counting method simply lacks the power of capturing context, irony, sarcasm, or the multiplicity regarding meanings of word. Moreover, the heavy reliance on the predetermined psychological constructs also poses limits in terms of the words being complied in the repository [11, 27]. Nevertheless, the word-count approach remains the most popular methodology across social science disciplines, primarily due to its high reliability in handling large corpora [24, 28, 29].

In response to the increasing demand for more efficient ways of analyzing large amount of texts and the development of computer programming in the past two decades, researchers have developed computerized programs tapping into both strategies outlined above. For instance, a program based on the Gottschalk-Gleser method of content analysis was applied to initial psychiatric screenings, although the reliability of the program raised certain concerns. Other word-based programs (such as the General Inquirer) proved useful for general analytic purposes [30] but lacked transparency with regard to its algorithms [24].

To be fair, recent years have seen an array of emerging tools, such as algorithmic coding software (mostly proprietary) and Unsupervised Topic Modeling [11, 31] being proposed and adopted in various fields. These tools, albeit extremely cost-efficient and versatile in certain analytic settings, run into the problems of lacking sufficient details regarding algorithm development, limited use for task- and theory-specific research, and/or requiring highly structured datasets [31, 32]. For these reasons, machine-learning techniques were not pursued in this project.

In an effort to improve the transparency, efficiency, and accuracy of text analysis, Pennebaker and colleagues [12, 33] developed a now widely-used software named Linguistic Inquiry and Word Count (LIWC). As a word-count program, the key component of LIWC is its embedded dictionary. LIWC processes individual or multiple textual files by searching and counting words that are listed in the pre-designated dictionary. The dictionary itself has been revised and validated over the course of two decades, and the most recent version consists of 6,400 English words/word stems, covering a range of social and psychological constructs such as affect, cognition, and biological processes [28]. Currently, LIWC is one of the most popular and reliable programs for text analysis available; it has been employed in hundreds of studies across the social sciences, including psychology, sociology, communication, political sciences, and economics [34, 35].

A particular advantage of LIWC in comparison to other text analysis programs is its versatility. It allows users to analyze texts based on dictionaries of their own interest. In other words, researchers can use LIWC for any analytical purposes as long as they have a validated dictionary extension. In that regard, the Moral Foundation Dictionary (MFD) is perhaps one of the most noteworthy examples. Developed by social psychologists Jesse Graham and Jonathan Haidt, the Moral Foundation Dictionary is a LIWC-enabled dictionary for measuring five moral considerations expressed in textual messages based on moral foundation theory [36]. Similar to the original LIWC dictionary, the MFD has been applied in other areas of research as well [37].

Due to its ease of use and the wide acceptance among social scientists, we pursued to construct a dictionary that is compatible with LIWC for the purpose of analyzing self-transcendent emotions in textual messages. The following sections discuss the methodological procedures we followed to develop and validate the resulting Self-Transcendent Emotion Dictionary (STED).

## Method

### Dictionary construction

The construction of the Self-Transcendent Emotion Dictionary (STED) was guided by the procedures utilized to develop the latest version of the LIWC dictionary (LIWC 2015) [28] and the MFD [37], relying on expansive and contractive steps comparable to those used to construct the latter. Specifically, in the first step, we identified words, word stems, phrases, associations, and synonyms within six categories: awe, admiration, elevation, gratitude, hope, and inspiration. Sources for the first-step procedure included official English dictionaries (*Merriam-Webster Dictionary*, *Roget’s Thesaurus*, *Collins Dictionary*, *Oxford English Dictionary*), online sources (transcripts from the 20 “most inspiring” TED talks: https://bit.ly/2UYZhmk), and open-ended responses to national and regional survey items on self-transcendent media [2, 9].

Step 1 resulted in the identification of 755 full words, word stems, and multi-words phrases (e.g., profound, inspire*, look forward, etc.). An additional 28 supplemental words/word stems were generated via a brain-storming session involving the authors and another senior researcher. Notably, there are a number of existing dictionaries that tap into certain constructs reflected in the STED [14, 32, 38, 39]. These lexicons, despite not being specifically consulted during our dictionary construction phase, were largely included in the 783 base words/phrases for future examination.

In Step 2, the authors examined each word in the dictionary in terms of their “fitness” for each category. In order for a word to remain in a given list, a majority of researchers had to agree on its inclusion. In case of disputes, we followed Pennebaker and colleagues’ [28] instruction and consulted additional sources to more precisely determine the words’ meaning and typical use to help decide whether to keep or remove the word from the dictionary. This procedure eliminated 141 words.

In Step 3, we checked the base rate of each word’s use within a wide range of texts, with the goal of ensuring that each word in the dictionary was in fact commonly used in daily spoken and written language. In this step, we uploaded the 614-item raw dictionary into a word count program used by Graham, Haidt, and Nosek [36] and checked the rate of each word used in a corpus consisting of 1,250 TED talk transcripts, 35 music, art, science, and technology books from Project Gutenberg (http://www.gutenberg.org/), more than 200,000 tweets collected for other research purposes over the course of three years, as well as the top 20 most shared *New York Times* articles for each day of February 2016. We removed from the dictionary all words that appeared 0 times across all of those sources and further scrutinized words that appeared fewer than 10 time. The resulting Self-Transcendent Emotion Dictionary included a total of 370 words, word stems, and (15) phrases reflecting six discrete emotional constructs: awe, admiration, elevation, gratitude, hope, and general inspiration.

Table 1 reports the numbers of lexicons in each of the six constructs and provides sample words for each category. Readers will note that the six emotion categories are not evenly represented in terms of the numbers of items; however, this should not be interpreted as one construct being more prevalent or important than the others but as a natural occurrence of language. Further, the sum of terms in each emotion category exceeds the total word count (i.e., 370) of the dictionary; that is because some words/word stems are cross-listed. For instance, “remark*” appears in both the awe and admiration categories; similarly, “promis*” is listed in both the elevation and hope categories.

**Table 1:** Summary of the initial version of the self-transcendent emotion dictionary.

Category	Number of Words	Samples
Awe	64	astonish*; awe*; beaut*
Admiration	110	belove*; bravery; think highly of
Elevation	53	prestige*; touch*; uplift*
Gratitude	64	charit*; happiness; selfless*
Hope	101	dream; embolden*; newborn
General/Inspiration	88	accomplish*; brought to tears; creative*

Total words and word stems in the dictionary = 370, including 15 phrases.

### Dictionary validation rationale

When it comes to the procedure of validating dictionary tools, as Pennebaker and colleagues [28] noted, “it’s tricky.” In their initial validation study, the researchers asked a group of judges to rate student essays on various dimensions represented in the LIWC Dictionary [33]. Then, the same essays were analyzed using LIWC. The validity of the dictionary was demonstrated by correlating the judges’ ratings with the LIWC results. Further validation was shown by correlating the LIWC analysis of essays with variables such as the Big Five personality traits [27]. In order to better delineate the concepts and evaluate the value of the key constructs of interests in those newly developed LIWC dictionaries, Donohue and colleagues [40] proposed and evaluated a three-step validation approach, which included (a) recruiting a group of individuals (undergraduate students) to evaluate the extent to which each word fit into all proposed constructs, (b) inviting subjects to write an essay tapping into the specific construct and applying the refined dictionary to the analysis of the essays to determine if the essays actually manifested with a higher percentage of words on the designated construct, and (c) training coders to code individual utterance/thought unit in each corpus and correlate the results with the LIWC analysis. By applying these strategies in the examination of their dictionary tool, Donohue, Liang, and Druckman [40] suggested that while the first two steps were necessary for establishing the face and construct validity of a dictionary, the third step may not be suitable for any validation scenario. They reasoned that when the proposed dictionary constructs are relatively abstract (i.e., lacks specific behavioral referents), human coders tend to look more broadly for extra contextual information and impressions to make their judgment on any given utterance. As a result, the dictionary for those constructs would appear to be somewhat limited compared with human judgments, generating false negatives. Put more bluntly, Donohue and colleagues noted that “the three-pronged validation approach may be too conservative for most purposes.” (p. 298).

Considering the advantages the previous studies and the locus of our dictionary constructs (i.e., human emotions), the current project adapted Donohue and colleagues [40] three-step validation approach with slight modifications: First, we replicated Step 1 and Step 2 of Donohue and colleagues for the purposes of establishing face validity and discriminant validity of the STED. Second, instead of examining specific paragraphs or utterance within individual text narrative, we applied the refined dictionary to the analysis of full news stories from the *New York Times*. The results of which were then contrasted based on the presence and absence of general “inspirationality” (a holistic sense of self-transcendence) judged by human coders. We contend that if the news articles judged to be inspirational contained more self-transcendence-related words across each individual construct—as listed in the STED and as compared to articles judged to be non-inspirational—it would lend support to the external validity and applicability of our dictionary tool.

The following sections detailed the validation efforts. Parts of the research that involved human subject were approved by the Marist Institutional Review Board (ref #F17-006).

## Results

### Validation step 1

As noted earlier, the purpose of our first validation step is to ensure words/phrases included in each construct possess face validity. To that end, independent raters with a diverse range of language habits were needed to cross-examining the fitness of each lexicons.

Specifically, a total of 82 communication undergraduates (*M*_*age*_ = 19.66) were recruited from a private university in the northeast region and a large public university in the northwest region of the U.S. To minimize participants’ research fatigue, raters were randomly assigned to rate each word/lexicon within one of the six constructs represented in the STED (as opposed to rate the dictionary in its entirety), rendering on average 80 words for each rater. Through an online Qualtrics survey, participants judged on a 5-point Likert-type scale, the extent to which each term reflected the definition of the respective construct, based on a description derived from the *Oxford English Dictionary* (*OED*), in exchange for a nominal amount of extra credit in their course. As an example, the definition of awe reads “Dread mingled with veneration, reverential or respectful fear; the attitude of a mind subdued to profound reverence in the presence of supreme authority, moral greatness or sublimity, or mysterious sacredness. Immediate and active fear; The feeling of solemn and reverential wonder, tinged with latent fear, inspired by what is terribly sublime and majestic in nature.”

When it comes to data reduction strategies, we considered mean, t-test, ratio, and factor analyses as utilized by Donohue and colleagues [40]. Given their findings suggested that the mean analysis consistently produced effective results in each of their validation steps, we then decided to proceed with mean reduction technique. In practice, words that had a mean fitness of 2.5 or lower were eliminated from the list of terms for the corresponding construct. As a result, a majority of the terms from the original STED were retained: *n* = 351 unique words (or 445 with duplicates/cross-listing; including 13 phrases); 32 lexicon and/or phrases were removed from the corresponding construct(s). Sample terms that were removed include: ennobl* (for general inspiration), titan* (awe), cater to (gratitude), foster* (elevation), venera* (admiration), and audaci* (hope).

To ensure that the mean reduction technique adequately functioned in the early validation stage, we also examined the internal consistencies of the final words and phrases within each construct as well as the correlations among each construct. The data subjected to analysis included individual ratings about the retained 445 words and phrases, with the number of words ranging between 59 (awe) and 103 (admiration) in each category. Results yielded high reliability, with Cronbach’s alpha ranging from .98 (elevation) to .99 (hope) and a mean correlation of .83 between constructs (see S2 Table for detail). Moreover, the reliability statistics remained intact with further item/word removals in each of the six constructs, which further supported the consistency and stability of the lexicons.

### Validation step 2

The first phase of our validation had independent judges to evaluate the face validity of the dictionary. The second phase aimed to test the extent to which the newly constructed tool would allow researchers to differentiate each of the six constructs. To achieve that goal, we followed a similar approach as previously discussed [27, 40] and recruited 210 participants to provide open-ended essays for each of the six constructs via Amazon’s Mechanical Turk (M-Turk) [41]. Upon giving their informed consent via the Qualtrics link, participants were randomly provided an *OED* definition of one of the six constructs and instructed to write a related essay over the course of a 10-minute period. We did not limit the topic or context about which the subjects should write their essays; rather, they were provided a few possible directions: “You may approach it in a free-flowing manner. For instance, you could discuss your interpretation of that emotion, your experience of it, how does that make you feel, what prompts you to feel that way, etc.” Presumably, due to our lenient demand on the M-Turk participants’ historical “approval rate” (i.e., > 60%), a number of participants (n = 40) provided inattentive or bogus responses, including plagiarized and/or irrelevant writings. These cases were therefore rejected, rendering 170 valid essays with word counts ranging from 21 to 851 words (*M* = 172.72, SD = 118.97). Authors of the approved essays were each rewarded $1 for their time.

Table 2 shows the average percentage of words captured by the refined dictionary from Step 1. Overall, when participants were instructed to write an essay about one of the six self-transcendent emotions, the STED identified a higher percentage of words associated with that construct than words associated with any other constructs in all cases, except one. For instance, 7.21% of words in gratitude-focused essays were listed in the gratitude section of the STED; this proportion was higher than the proportion of words associated with any other construct in the STED. Furthermore, the differences observed were statistically significant in five out of the six cases (see t-test results in Table 2).

**Table 2:** Percentage of terms for each self-transcendent construct as identified in construct-themed essays.

	Essay Themes											
	General		Awe		Gratitude		Elevation		Admiration		Hope	
	Mean	SD	Mean	SD	Mean	SD	Mean	SD	Mean	SD	Mean	SD
General	1.42	1.03	0.79	2.15	0.47	0.51	0.75	1.24	0.87	1.89	0.63	0.64
Awe	4.81	2.62	6.12	5.19	4.40	1.78	1.00	1.13	6.09	4.22	0.36	0.63
Gratitude	0.82	0.84	1.81	2.11	7.21	3.45	1.24	1.68	5.99	4.13	0.75	1.12
Elevation	4.26	2.73	0.75	0.92	4.94	2.54	3.60	2.64	1.06	1.06	4.83	2.50
Admiration	5.10	2.64	5.74	4.23	5.75	2.36	1.03	0.98	7.12	5.23	0.76	0.94
Hope	5.24	3.20	1.42	1.60	1.18	0.97	4.04	2.49	1.59	1.55	7.65	3.61
*t*^†^	3.73*		7.54*		7.00*		1.00		3.47*		7.07*	
*d*	.56		.63		1.52		.21		.89		1.62	

Shaded cells highlight the percentage of words associated with a construct within essays written about that construct, thus comparisons within construct should be drawn vertically.

^†^ *t*-tests were performed within each construct (across columns) with the corresponding dictionary dummy coded as 1, and the others coded as 0.

* *p* < .001.

The one instance where significance was not observed involved the elevation construct. In elevation-themed essays, the dictionary identified similar percentages of elevation (3.60%) and hope terms (4.04%). However, in hope-themed essays, the STED identified more hope (7.65%) than elevation terms (4.83%). Thus, we contend that the findings tend toward discriminant validity between these two constructs, as well as provide strong support for discriminant validity between all others. One other notable observation concerns the fact that the general inspiration-themed essays did not result in descriptively higher percentage of inspiration terms (1.42%) in contrast with other emotional terms (e.g., admiration 5.1%; awe 4.81%; hope 5.24%). Nevertheless, this was deemed acceptable given the general inspiration category represents broader dimensions of transcendence, ones that clearly overlap with the specific self-transcendent emotions in question [17, 23].

In sum, results from Step 2 indicated that, by and large, the lists of lexicons involved possess reasonable level of construct validity. The relative insensitivity of elevation terms (as compared with hope terms), albeit conceivable in theory, calls for caution and further investigation; therefore, we proceeded with validation step 3, which applies the dictionary into common media writings, namely news articles.

### Validation step 3

As noted earlier, the primary goal of validation step 3 was to establish the external validity and applicability of this newly constructed tool. That is, we aimed to show that the dictionary is functional when applied to general texts as generated and consumed in daily lives (as opposed to essays written on-demand as in Step 2). To that end, we trained human coders to judge whether popular *New York Times* stories were “inspirational” or not. The test of the validity of our dictionary tool would be to demonstrate that news articles judged to be inspirational would contain more words listed in the STED than stories judged as non-inspirational. Note that the binary human judgment of the stories’ inspirational nature was considered a comprehensive assessment of the self-transcendent emotions, as coding the presence and absence of each emotion in textual narratives is practically challenging, if not impossible.

To collect the news articles for analysis, we designed a project-developed web crawler that utilized the *New York Times* API to systematically scrape the Top 20 most emailed, most tweeted (on Twitter), and most posted to Facebook lists at four different times each day (i.e., every six hours) over a 3-month period of time (February to April 2016). The web crawler captured each article’s title, author, publication date, section, rank (1–20), URL, summary, and full text. During our sampling period, a total of 44,306 articles were captured. After deduplication and removing articles that contained missing data, 3,885 unique articles remained. This final sample (*n* = 3,885) was subject to human coding and computerized analyses using the refined STED.

The lead author and two trained graduate student coders were involved in the process of coding the articles as either inspiring or not. Due to the large number of news articles, a random sample of 10% of the articles from each of the three datasets (i.e., most emailed, tweeted, and posted to Facebook) was generated for practice coding and to establish intercoder reliability. Before the reliability test, the lead author fully trained the coders on the binary coding scheme, followed by a practice coding session. Results were discussed, and disputes were reconciled. Then, coders received the title, author, summary, and full texts of the articles and proceeded to code the articles to establish reliability. Final intercoder reliability for inspirational nature was Krippendorf’s *α* = .91 (with 94% agreement). Given the sufficient reliability, each coder then assessed one-third of the full sample. As a result (see Table 3), 861 (22.17%) articles were judged as inspirational, while the rest were categorized as non-inspirational.

**Table 3:** Summary of self-transcendent word use in socially shared *New York Times* articles.

	Inspirational stories (n = 861)		Non-inspirational stories (n = 3,024)			
	M	SD	M	SD	*t*	*d*
STED words total	2.15	1.0	1.84	.93	8.28	.25
Awe-oriented words	.47	.42	.37	.37	6.63	.25
Gratitude-oriented words	.48	.39	.44	.64	1.87	.08
Elevation-oriented words	.37	.31	.31	.33	4.91	.19
Admiration-oriented words	.66	.53	.54	.48	6.24	.24
Hope-oriented words	.73	.46	.67	.42	3.74	.14
General inspiration-oriented words	.41	.34	.33	.36	5.73	.23

Per LIWC, all analyses were conducted on percentages of words present; thus, the appropriate values herein represent such. All observed differences were significant at the *p* < .01 level.

Then, the full text of 3,885 articles was analyzed via LIWC 2015 using the refined STED. The output of the program showed the percentages of all dictionary words, as well as the number of words present in each article for the six categories. An independent sample *t*-test suggested that stories judged to be “inspirational” did, in fact, contain a higher percentage of STED words than non-inspirational stories. We interpret this as initial evidence that the STED is practically valid (Table 3; *t* = 5.73, *p*_*one-tailed*_ < .001, Cohen’s *d* = .25). Additional *t*-tests revealed that inspirational stories had significantly higher percentages of words associated with all six constructs in the STED as compared with non-inspirational stories, further lending validation to the dictionary. S1 Table shows the 10 most frequently used terms in each of the six STED constructs across the sample of articles.

Admittedly, the observed effects sizes appear to be relatively small, though they are in line with prior studies utilizing word-count based dictionaries [34]. In fact, the average effects sizes (*d* = .19) observed in the validation of the STED are (descriptively) higher than what was reported in previous research about gender differences of language use (i.e., *d* = .14) [34], an area which had been extensively examined with adequate sample sizes and effect sizes were readily available [42]. Moreover, the *New York Times* stories used for our validation purpose are already highly transmitted among readers (top 20s). The popularity of the texts might imply certain homogeneity in their writing style, authors’ background, and the readership each story would appeal to. If that was the case, the effect sizes observed in this validation step could be higher among general writings which vary across individuals, geographic regions, socio-economic backgrounds, and (sub)cultures. For these reasons, we contend that the differences in self-transcendent language use between inspirational and non-inspirational stories as observed herein are not only statistically significant but also conceptually meaningful.

## Discussion

The overarching goal of this study was to develop a computational tool that can enable researchers to analyze inspirational or self-transcendent emotions expressed in (and perhaps elicited by) textual media content. Through a brief review of text analysis literature, we identified LIWC—a dictionary-based text analysis program—as a viable platform where we could build such a tool as an extension. Consequently, we developed the Self-Transcendent Emotion Dictionary (see S1 Text), which in its refined version consists of 351 commonly used English words, word stems, and phrases listed associated with six categories related to self-transcendent emotions. The dictionary tool was validated following a modified procedure recommended by Donohue, Liang, and Druckman [40]. Namely, individual words were rated with regard to their fitness into the proposed construct, and then used to analyze essays written to reflect that construct. To ensure its external validity and applicability, the refined dictionary was uploaded into LIWC to examine the word used in a set of *New York Times* articles that were first human-coded based on their inspirational nature. As expected, we found that inspirational stories contained significantly higher percentages of words in the than non-inspirational stories, both in overall terms and within each of the six emotion-based categories. Thus, we contend that the refined STED does seem to carry adequate levels of face, construct, and external validity.

With this groundwork, researchers are equipped with initial tools to examine inspirational media content or self-transcendent emotions in various text formats, including speech transcripts, books, news articles, movie dialog, (text-based) social media posts, and lyrics. For instance, a recent study [43] investigated the use of positive words in books and news stories published in the United States since the 1800s. Similar research could be conducted with STED from a variety of theoretical bases. From our perspective, it is theoretically intriguing to examine the use of inspirational words in combination with data concerning individuals’ actual behaviors. Such inquiry might help to provide further evidence to the presumed relationships between self-transcendent emotions and behavioral. However, caution should be applied when using the dictionary for single texts or corpora made out of short texts (e.g., tweets; more on that later).

On a methodological level, a particular challenge that researchers in mass communication and journalism constantly battle against is designing effective stimulus materials that can adequately evoke a certain emotional experience in question. As the field moves to probe the causal effects of discrete emotional appeals (in addition to content valence) on audiences’ perceptions and attitudes, researchers might find themselves in need of concrete guidelines for designing and soliciting media stimuli. An existing maneuver is to manipulate the narrative outcomes of specific instance presented in the stimuli [44]. The problem, however, is that such manipulations tend to produce mixed emotions rather than pure emotions. What our dictionary tool offers, from our perspective, is a set of lexicons which researchers could adopt in their stimuli design without the necessity of severely altering the narrative outcomes. As such, researchers interested in the effects of self-transcendent media experience are better equipped to design narrative-based content that actually leads to awe, admiration, elevation, gratitude, and/or hope.

Another promising direction might be to apply the STED in the analysis of text-based social media posts. Granted, a social media post with an average length of 50 to 100 words might not always contain words in our dictionary, and calculating a score based on the occurrence of a couple of words (within a single post) might be limited. Thus, the application of a dictionary tool like ours would demand creative ways of data aggregation in specific research scenarios. For example, in one study which examined the live tweets sent during the Season 3 finale of *Downtown Abbey* [37], researchers were able to look into the morally oriented nature of live tweets according to the characters featured in the TV show. Similarly, as the marketing and public relation industries increasingly recognize the merits of self-transcendent emotions, applying our dictionary to gain consumer insights concerning brands or companies on social media is foreseeable.

Finally, modern information system designers might find our dictionary valuable in creating recommendation systems for various purposes. For instance, companies that possess large volumes of data and digital content such as YouTube or Steam nowadays often need to respond to users’ demand for high-quality videos or games that are meaningful and inspiring. By incorporating STED into their content recommendation algorithms, these companies are better equipped to supply enriching content for their users.

Despite the wide potential of the STED as a new text analytic tool for psychologists and communication researchers, a few caveats are in order. First, the dictionary was construct based on the conceptual scheme of self-transcendent emotions. Hence, new categories should be taken into consideration as researchers continue gaining understanding about inspirational media content and self-transcendent emotions. Similarly, one must not consider the STED as containing an exhaustive list of words, word stems, or phrases under each of the six constructs; rather, it contains words that are *commonly* used in people’s daily spoken and written language. Therefore, given the ever-changing nature of daily language use, continuing to refine the dictionary is a must. In that vein, this dictionary (as with any other dictionary of its kind) relies heavily on the initial corpus of texts; as such, the linguistic styles of *New York Times* articles, TED talks, and college students’ responses surely exerted an influence on the foundation of the dictionary. Nonetheless, we believe that foundation to be strong, considering the diversity of the seminal data as well as the additional steps used to ensure the breadth of the initial word list. Moreover, although the validity of the dictionary was demonstrated among student essays and the most shared *New York Times* articles, the validation of STED in other contexts is warranted.

The last limitation pertains to the general concerns with word-count/dictionary-based tools as a whole. These programs often fall short in analyzing naturally occurring language while accounting for the rhetorical techniques (e.g., irony, analogy, sarcasm) being used, as well as the multiple meanings of words [12, 28, 45]. Certain word stems inevitably come with unintended manifestations (e.g., “promiscuous” as in “promis*,” and “touchy” as in “touch*). Similarly, phrases such as “give rise to,” “brought to tears,” and “think highly of” are confined to their current tense/form. As such, efforts for continuous refinement and addition to this dictionary are encouraged, if not necessary. At this point, we are cautiously optimistic about the long-term potentials of this tool, particularly when it comes to analyzing large sets of data. Nonetheless, we believe researchers would benefit from utilizing this tool in combination with certain levels of human judgment.

More than anything, we hope that this research will be useful to those who seek to explore self-transcendent emotions in textual narratives. As this content is increasingly popular, available, and consumed, it is imperative for social scientists, particularly psychologists and communication scholars, to continue to understand how such content can contribute to increased human connectedness, prosociality, human flourishing, and well-being [46]. We hope that our tool can motivate and pave the way for more research in this area.

## Supporting information

S1 Table Frequently used terms in the most socially shared *New York Times* articles. (DOCX)

S2 Table Factor correlation matrix. (DOC)

S1 Text The self-transcendent emotion dictionary. (DIC)
