# Supplementary material for: Developing and validating the self-transcendent emotion dictionary for text analysis
Source: PLoS One. 2020 Sep 11;15(9):e0239050. doi: 10.1371/journal.pone.0239050 (PMC7485772; doi:10.1371/journal.pone.0239050)
Supplement: S1 Table — (DOCX) [file pone.0239050.s001.docx]

| Table. *Most Frequently Used Terms by Construct in Socially Shared New York Times Articles.* | | | | |
| --- | --- | --- | --- | --- |
|  | All Stories | | Inspiring Stories | |
|  | Popular terms | Frequencies | Popular terms | Frequencies |
| Awe | power* | 3,762 | lov* | 588 |
|  | lov* | 2,785 | power* | 376 |
|  | great | 2,516 | great | 347 |
|  | interest* | 2,394 | interest* | 263 |
|  | christ* | 1,987 | god* | 204 |
|  | grand* | 1,532 | wonder* | 177 |
|  | religi* | 1,133 | grand* | 176 |
|  | wonder* | 1,123 | inspir* | 170 |
|  | remark* | 1,079 | christ* | 165 |
|  | god* | 962 | beaut* | 159 |
| Admiration | help* | 5,700 | help* | 781 |
|  | kind* | 2,736 | kind* | 366 |
|  | favor* | 1,632 | favor* | 174 |
|  | earn* | 1,414 | recogn* | 147 |
|  | acknowledg* | 1,014 | happy | 130 |
|  | recogn* | 957 | earn* | 116 |
|  | thank* | 872 | virtu* | 114 |
|  | credit | 831 | thank* | 99 |
|  | pleas* | 784 | acknowledg* | 97 |
|  | virtu* | 698 | pleas* | 93 |
| Elevation | hope* | 2,177 | hope* | 276 |
|  | promis* | 1,225 | encourag* | 141 |
|  | encourag* | 985 | advanc* | 124 |
|  | advanc* | 903 | moving | 115 |
|  | warm* | 805 | promis* | 88 |
|  | moving | 753 | moral* | 82 |
|  | touch* | 702 | touch* | 82 |
|  | moral* | 675 | faith* | 80 |
|  | strength* | 644 | warm* | 80 |
|  | faith* | 610 | strength* | 62 |
| Gratitude | valu* | 1,637 | artist* | 233 |
|  | fall | 1,223 | valu* | 169 |
|  | perfect* | 905 | achiev* | 156 |
|  | artist* | 868 | perfect* | 128 |
|  | approv* | 745 | hero* | 119 |
|  | achiev* | 723 | enjoy* | 111 |
|  | prefer* | 702 | passion* | 90 |
|  | enjoy* | 673 | prefer* | 84 |
|  | hero* | 643 | joy* | 78 |
|  | highly | 576 | vast | 77 |
| Hope | belie* | 3,786 | learn* | 395 |
|  | grow* | 3,393 | grow* | 381 |
|  | secur* | 2,752 | belie* | 351 |
|  | learn* | 2,316 | expect* | 261 |
|  | expect* | 2,261 | future | 196 |
|  | future | 1,504 | opportunit* | 138 |
|  | opportunit* | 1,075 | secur* | 134 |
|  | trust* | 923 | comfort* | 103 |
|  | forward | 813 | dream | 97 |
| General/Inspiration | fun* | 4,150 | fun* | 443 |
|  | better | 3,076 | better | 423 |
|  | care | 2,302 | care | 323 |
|  | success* | 2,174 | success* | 310 |
|  | heart* | 1,419 | energ* | 225 |
|  | imagin* | 1,257 | imagin* | 192 |
|  | influen* | 1,055 | heart* | 187 |
|  | energ* | 1,038 | influen* | 157 |
|  | expand* | 714 | creativ* | 136 |
