# Supplementary material for: Developing and validating the self-transcendent emotion dictionary for text analysis
Source: PLoS One. 2020 Sep 11;15(9):e0239050. doi: 10.1371/journal.pone.0239050 (PMC7485772; doi:10.1371/journal.pone.0239050)
Supplement: S2 Table — (DOC) [file pone.0239050.s002.doc]

| *Table.* Correlations matrix for STED constructs | | | | | | |
| --- | --- | --- | --- | --- | --- | --- |
|  | Inspiration | Awe | Gratitude | Elevation | Admiration | Hope |
| Inspiration | 1 | — | — | — | — | — |
| Awe | .96** | 1 | — | — | — | — |
| Gratitude | .90** | .73** | 1 | — | — | — |
| Elevation | .79** | .74** | .82** | 1 | — | — |
| Admiration | .80** | .80** | .86** | .82** | 1 | — |
| Hope | .92** | .71^**^ | .87** | .86** | .82^**^ | 1 |

*Note.* ** Correlation is significant at the 0.01 level.
